# Assessment of drugs administered in the Middle East as part of the COVID-19 management protocols

**DOI:** 10.1007/s10787-022-01050-7

**Published:** 2022-08-26

**Authors:** Engy Elekhnawy, Walaa A. Negm, Suzy A. El-Sherbeni, Ahmed Zayed

**Affiliations:** 1 Pharmaceutical Microbiology Department, Faculty of Pharmacy, Tanta University, Elguish Street (Medical Campus), Tanta, 31527 Egypt; 2 Pharmacognosy Department, Faculty of Pharmacy, Tanta University, Elguish Street (Medical Campus), Tanta, 31527 Egypt; 3 Institute of Bioprocess Engineering, Technical University of Kaiserslautern, Gottlieb-Daimler-Straße 49, 67663 Kaiserslautern, Germany

**Keywords:** Antiviral, COVID-19 pandemic, Egypt, Middle East, Protocol, Saudi Arabia

## Abstract

The pandemic spread of coronavirus (COVID-19) has been reported first at the end of 2019. It continues disturbing various human aspects with multiple pandemic waves showing more fatal novel variants. Now Egypt faces the sixth wave of the pandemic with controlled governmental measures. COVID-19 is an infectious respiratory disease-causing mild to moderate illness that can be progressed into life-threatening complications based on patients- and variant type-related factors. The symptoms vary from dry cough, fever to difficulty in breathing that required urgent hospitalization. Most countries have authorized their national protocols for managing manifested symptoms and thus lowering the rate of patients’ hospitalization and boosting the healthcare systems. These protocols are still in use even with the development and approval of several vaccines. These protocols were instructed to aid home isolation, bed rest, dietary supplements, and additionally the administration of antipyretic, steroids, and antiviral drugs. The current review aimed to highlight the administered protocols in the Middle East, namely in Egypt and the Kingdom of Saudi Arabia demonstrating how these protocols have shown potential effectiveness in treating patients and saving many soles.

## Introduction

Following the Spanish flu (H1N1) in 1918, Asian flu (H2N2) in 1957, Hong Kong flu (H3N2) in 1968, and Pandemic flu (H1N1) in 2009, the World Health Organization (WHO) considered officially on the 11th of March 2020 that the symptoms associated with the current coronavirus as the fifth viral pandemic disease. The virus was first isolated from a novel human pneumonia case in Wuhan, China as a novel zoonotic disease in Dec. 2019, and hence, named coronavirus disease or COVID-19 (Dong et al. 2020). Its outbreaks have resulted in generalized chaos of human life since this date on Earth and a huge number of deaths all over the world. The WHO estimates the global case fatality rate by 2.2% for COVID-19 (Marois et al. 2020). The WHO confirmed that there was evidence that the novel coronavirus may be transferred from human-to-human, *i.e.*, droplets infection, causing the severe respiratory syndrome, which is a critical danger to public health, and therefore, was named also severe acute respiratory syndrome coronavirus-2 (SARS-CoV-2) (Zheng 2020). Spreading of COVID-19 in 223 countries with more than 472 million cases, and 6 million deaths, was reported by WHO till March 2022. The recent SARS-CoV-2 variant, Omicron has been reported in 76 countries since November 2021(Cascella et al. 2022).

Previous literature has classified coronaviruses into alpha, beta, gamma, and delta types, *i.e.*, four sub-groupings. SARS-CoV-2 (a beta virus) has some characteristics differentiating it from most of the past decade's pandemics as SARS-CoV-1. First and foremost, its rate of transmission is very high as its basic reproduction number (R0) ranges from 1.9 to 6.5 (Li et al. 2020; Park et al. 2020a, b), *i.e.*, the virus is contagious to a large extent and can spread very widely in a very short period (Peeri et al. 2020). Its high infectivity is mainly attributed to its ability to be transmitted from both asymptomatic and pre-symptomatic cases (Bai et al. 2020). Moreover, the virus has demonstrated further fatal variants *via* several genomic mutations, including the UK B.1.1.7 strain, P.1 or B.1.1.248, 1.351, B.1.526, and B.1.427/B.1.429. These variants have worsened subsequently the crisis. The virus life cycle and mode of infection have been discussed previously in several publications and the readers can refer to for further information (V’kovski et al. 2021).

Besides, the spectrum of its severity is different. Most of the cases infected with COVID-19 manifest mild or no symptoms (Lake 2020), while in the remaining cases, the disease can worsen into life-threatening bilateral pneumonia, with symptoms varying from dyspnea to complete respiratory failure and death (Day 2020; Odone et al. 2020). Whereas most COVID-19 cases have mild (40%) or moderate (40%) symptoms, about 15% of cases develop severe sickness which needs oxygen support, and the remaining 5% develop critical disease (Surveillances 2020) as shown in Table 1 There are some reported risk factors for severe COVID-19 disease and death including old age, smoking, cancer, cardiac diseases, diabetes, hypertension, and chronic lung diseases (Alqahtani et al. 2020; Wang et al. 2020a, b, c; Organization 2020a, b; Zhou et al. 2020).

**Table 1:** Symptoms spectra of COVID-19 manifested in infected patients according to the disease severity

Degree of severity	Characteristics	References
Mild cases	-An uncomplicated viral infection in the upper respiratory tract -Non-specific symptoms like fever, fatigue, dry or productive cough, muscle pain, sore throat, anorexia, dyspnea, congestion, and headache -Possibility of nausea, vomiting, and diarrhea	(Chen et al. 2020a, b; Guan et al. 2020; Wang et al. 2020a, b, c)
Moderate cases	Patients show in addition to mild symptoms, -Clinical and/or radiographic evidence of lower respiratory disease, -Slighlty low oxygen saturation, i.e., ≥ 94%	(Gandhi et al. 2020)
Severe cases	In addition to the previously mentioned symptoms, -Oxygen saturation < 94%, respiratory rate ≥ 30 breaths/min, -lung infiltrates > 50%	
Critical cases	-Various complications like acute respiratory distress syndrome (ARDS), respiratory failure, sepsis, thromboembolism, -multi-organ failure	(Zaim et al. 2020)

To control the COVID-19 infection spread and to get into a normal post-pandemic situation, we need to raise the population's immunity. This can be achieved either naturally or *via* using vaccines (DeRoo et al. 2020). Using vaccines appears to be the quick fix to stop this pandemic spread, and thus they must be distributed very rapidly and efficiently. Consequently, the optimal method to rapidly control the current global crisis of pandemic COVID-19 is by developing and applying safe and effective vaccines (Shah et al. 2020). For the past few months, different vaccines have been implemented globally, and additional ones are under development, some of them have reached the application stages and others are in advanced clinical trials phases (Hagens et al. 2021). Based on the preliminary results of the current clinical trials of candidate vaccines, it seems that many vaccines can induce an adequate immune response and are generally well-tolerated but may be effective for a short period because of incomplete revealing of the genetic code of SARS-CoV-2 (Le et al. 2020). Thus far, they can protect against the serious symptoms of the disease and can potentially inhibit asymptomatic infections and the subsequent transmission (Jackson et al. 2020; Xia et al. 2020).

The development and implementation of the COVID-19 vaccine have been carried out under huge clinical, economical, and political pressure. Even with the dense need for superfast tracks for vaccine production, the Food and Drug Administration (FDA) took a decision not to authorize any vaccine without fulfilling its standards (Avorn and Kesselheim 2020). This is since any defect in the safety regulations for new vaccine authorization could potentially threaten the confidence and trust in the well-established global vaccination programs (Trogen et al. 2020). Thus, vaccines of COVID-19 have undergone very careful assessments for licensing, registration, and implementation, and this will continue to be applied to the new coming vaccines in 2021. Currently, several vaccines have been introduced to the market, including BioNTech-Pfizer (BNT162b2), Astra Zeneca (AZD1222), Moderna vaccine (mRNA-1273), Johnson & Johnson Vaccine (Ad26.COV2.S), and others, based on various technologies.

The use of treatment protocols has been encouraged since the development of an effective vaccine takes years (Haque and Pant 2020) and when available, the required doses are not enough for the world's countries’ populations. Hence, most of the world's countries have authorized the use of some conventional drugs since the beginning of the pandemic, based mainly on experiences acquired from previous coronavirus diseases, severity, and the response of the diverse patients to drugs.

The present article reviewed the official protocols used in the Middle East, especially in Egypt and the Kingdom of Saudi Arabia, where they are among the most important countries that have highly qualified health care systems and succeeded to face to a great extent the COVID-19 infection waves in the Middle East region (Hassany et al. 2020; Post et al. 2021). This review is a part of our ongoing project (Khalifa et al. 2020a, b) where the aim is to assess the current COVID-19 situation including the managing protocols, specifically the commonly used drugs in both countries as representative examples of the Middle East region. These points affect consequently choosing the best drug combinations and assessing their importance and mechanism of action to decrease and treat the symptoms effectively, in parallel with the world's plan to discover and develop new lines of treatments and vaccines.

## The pandemic COVID-19 and its waves

To date, the pandemic COVID-19 has experienced two waves in many countries. Nevertheless, a lot of world condition is suffering now from the third wave. The manifestations of fever, cough, dyspnea, and pneumonia were comparable in both waves, though frequent cases in the second wave presented with gastrointestinal symptoms such as nausea, vomiting, and abdominal pain (Zhang et al. 2020; Fan et al. 2021).

A noticeable difference between the first and second waves is the decrease in the case fatality rate (CFR) relative to the first one. CFR of COVID-19 is calculated by dividing the reported COVID-19 deaths number by the number of total cases. CFR is an important indicator for the quantification of the disease severity and the efficacy of treatment (Fan et al. 2021). There could be many reasons for this decrease in COVID-19 CFR in the second wave. First, a very large number of people in the vulnerable groups (such as the elderly and those with health conditions) probably died in the first wave, particularly in the countries with high infection rates. Second, the healthcare system capacity in many countries might have been better prepared for the second wave (Saito et al. 2020; Fan et al. 2021). Finally, the change of age structure of the infected people is a factor in the decrease of the CFR as the first wave involved mainly the elderly and those with health conditions while the second wave involved mainly children and healthier young individuals. The low compliance of social distancing and thus the increase in the virus transmission in children and young people could be a reason for this change in age structure in the second wave (Zhao et al. 2020). In early 2021 the vaccination campaigns started with a general hope to reach the end of corona tunnel. Lock-down measures were not fully respected in different countries, some relaxation was observed while spreading of more aggressive variants such as British, South-African and Brazilian variants. As a result, the third wave of COVID-19 appeared with increasing infection rates in different countries such as France, Germany as well as India which showed the highest peak in the third wave. USA and UK experienced a successful vaccination strategy (Rothengatter et al. 2021). The fourth and fifth waves were also experienced, which was especially affecting adolescents and young adults due to epidemic exhaustion, wrong information, absence of coordination among different administrative organizations, disagreements in many communities between public health services and the legislative actuality, and the appearance and distribution of the new SARS-CoV-2 variants (García-Basteiro et al. 2020; Han et al. 2020).

Now Egypt faces the sixth wave of the pandemic with controlled governmental measures. The majority of cases could be treated at home, and the severe cases are hospitalized. The World Health Organisation reported that the most likely causes of the recent increase in infections worldwide are two offshoots of the Omicron variant, BA.4 and BA.5 (www.thenationalnews.com).

## Demographics of Egypt and Saudi Arabia Kingdom and COVID-19 cases

Demographic studies revealed that Egypt and Saudi Arabia are quietly different. The current population, i.e., till July 17, 2022, of Egypt is 106,432,734. The population density in Egypt is 103 per Km^2^ (266 people per mi^2^). The total land area is 995,450 Km^2^ (384,345 sq. miles) and 43.0% of the population is urban (44,041,052 people in 2020). Furthermore, the percentage of elderly Egyptians (65+ years) is 4.44% of the total population. The median age in Egypt is 24.6 years (Worldometer 2022). While the current population of Saudi Arabia is 35,916,911. The population density in Saudi Arabia is 16 per Km^2^ (42 people per mi^2^). The total land area is 2,149,690 Km^2^ (830,000 sq. miles) and 84.0 % of the population is urban (29,255,576 people in 2020). The percentage of the Saudi elderly (65+ years) is 3.63% of the total Saudi population and the median age in Saudi Arabia is 31.8 years.

**Fig. 1:**
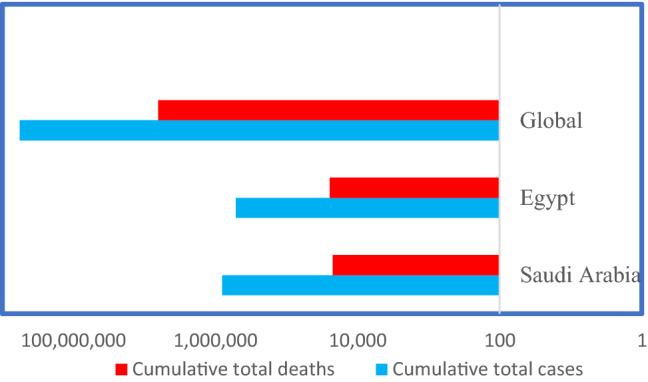
COVID-19 total cases and deaths recorded in Saudi Arabia and Egypt versus global cases recorded from 17th of July 2022 (Using logarithmic scale based 10)

WHO has reported till the 17th of July 2022 that the number of reported COVID-19 cases in Egypt is 515,645 (0.48 % of the total population), while in the Kingdom of Saudi Arabia is 803,158 (2,23 % of the total population). The cumulative total death cases in Egypt are 24,613 whereas in the Kingdom of Saudi Arabia are 22,362. These dates are obtained from the WHO Coronavirus (COVID-19) Dashboard, accessed on 17-6-2022 (WHO 2022). In Saudi Arabia, among closed cases that had an outcome 787,599 (98%) recovered and/or discharged and 22,362 (2%) deaths. While among 6,329 active cases (which are currently infected patients), 151 cases are considered critical. In Egypt, 122 cases are critical ones of 48,850 active cases (Worldometer 2022). COVID-19 total recorded cases versus total deaths in Egypt and Saudi Arabia are demonstrated in Figure 1 and Table 2.

**Table 2:** Demographics of COVID-19 cases in Egypt and the Kingdom of Saudi Arabia, based on WHO Coronavirus (COVID-19) Dashboard accessed on 17–07–2022

Country	Population	Coronavirus cases	Recovered	% Recovery	Deaths	Active cases	Serious or critical
Global	7,961,109,690	567,151,182	538,245,891	95%	6,386,967	22,518,324	38,987
Kingdom of Saudi Arabia	35,916,911	803,158	787,599	98%	22,362	6,329	151
Egypt	106,432,734	515,645	442,182	92%	24,613	48,850	122

## Kingdom of Saudi Arabia's COVID-19 preparedness and response: building on the MERS experience

It is noteworthy that the region of the Middle East had previously witnessed outbreaks of the two other coronaviruses, SARS, and the Middle East respiratory syndrome coronavirus (MERS). MERS is an emerging viral respiratory disease caused by the MERS coronavirus, also known as MERS-CoV, which was first reported in Saudi Arabia in 2012. Thus, the affected countries had subsequently learned some lessons about handling outbreaks (Mounts et al. 2013; Sawaya et al. 2020). For instance, the ministry of health in the Kingdom of Saudi Arabia established the command-and-control center and the Saudi center for disease control and prevention shortly after the detection of MERS. Today, these two centers represent the country's frontline response to COVID-19 (Malik and Mahjour 2015). Furthermore, the Saudi Ministry of Health developed the National Health Laboratory (NHL) as a reference laboratory with a focus on advanced diagnostics for infectious diseases in high biocontainment laboratories (Algaissi et al. 2020).

Saudi Arabia's Ministry of Health, military hospitals, and other government-sponsored hospitals offer free healthcare services to the public. There is also a large network of for-profit hospitals in the private sector throughout the world. The number of beds per 1000 people in Saudi Arabia is 2.2. Saudi Arabia is also a signatory to the WHO International Health Regulation (IHR; 2005) and has been reporting on pandemic preparedness and adhering to WHO policies on infection prevention and control (IPC) (Memish et al. 2014; Hashem et al. 2019). To meet the rising demand for health care services in Saudi Arabia, the Saudi Vision 2030 considers fundamental systemic changes in the healthcare sector (Algaissi et al. 2020). Biosafety in diagnostic laboratories has improved significantly, like the implementation of strict IPC systems in all hospitals throughout the world. In addition, the Saudi Ministry of Health has designated more than 25 regional hospitals to isolate and treat MERS patients. These hospitals are now fully equipped to handle COVID-19 patients and have begun to do so (Algaissi et al. 2020).

## Development of management protocols

It was a critical issue to prevent and control COVID-19 on the national and global scales owing to the intense increase in COVID-19 cases all over the world. The governments of each country provided guidelines to teach citizens how to protect themselves from COVID-19. Additionally, they provided important guidance for healthcare professionals to help them take steps to reduce the spread of COVID-19 (Yoo et al. 2020). Bearing in mind that each country had various health care systems, capacities, risks, socioeconomic and political challenges, it was predictable that each nation reacted to this global threat with relatively unique measures (Yoo et al. 2020).

The used protocols are mainly based on three main aspects. The first one is to apply transmission-based precautions, including isolation for symptomatic patients for 10 days after the onset of symptoms, plus three days (at least) without symptoms. The second aspect is the treatment of acute co-infections by using antibiotic therapy. The third aspect includes the prevention of complications (Organization 2020a, b).

It was hard to find out an optimal therapeutic compound that consistently resulted in positive results across SARS, MERS, and COVID-19. This might be because there is not a universal “cure” for such viral diseases. The very subtle differences among these three coronaviruses, along with the shortage of objective information from the clinical experiences of the previous SARS and MERS epidemics, might be other reasons (Han et al. 2021).

**Fig. 2:**
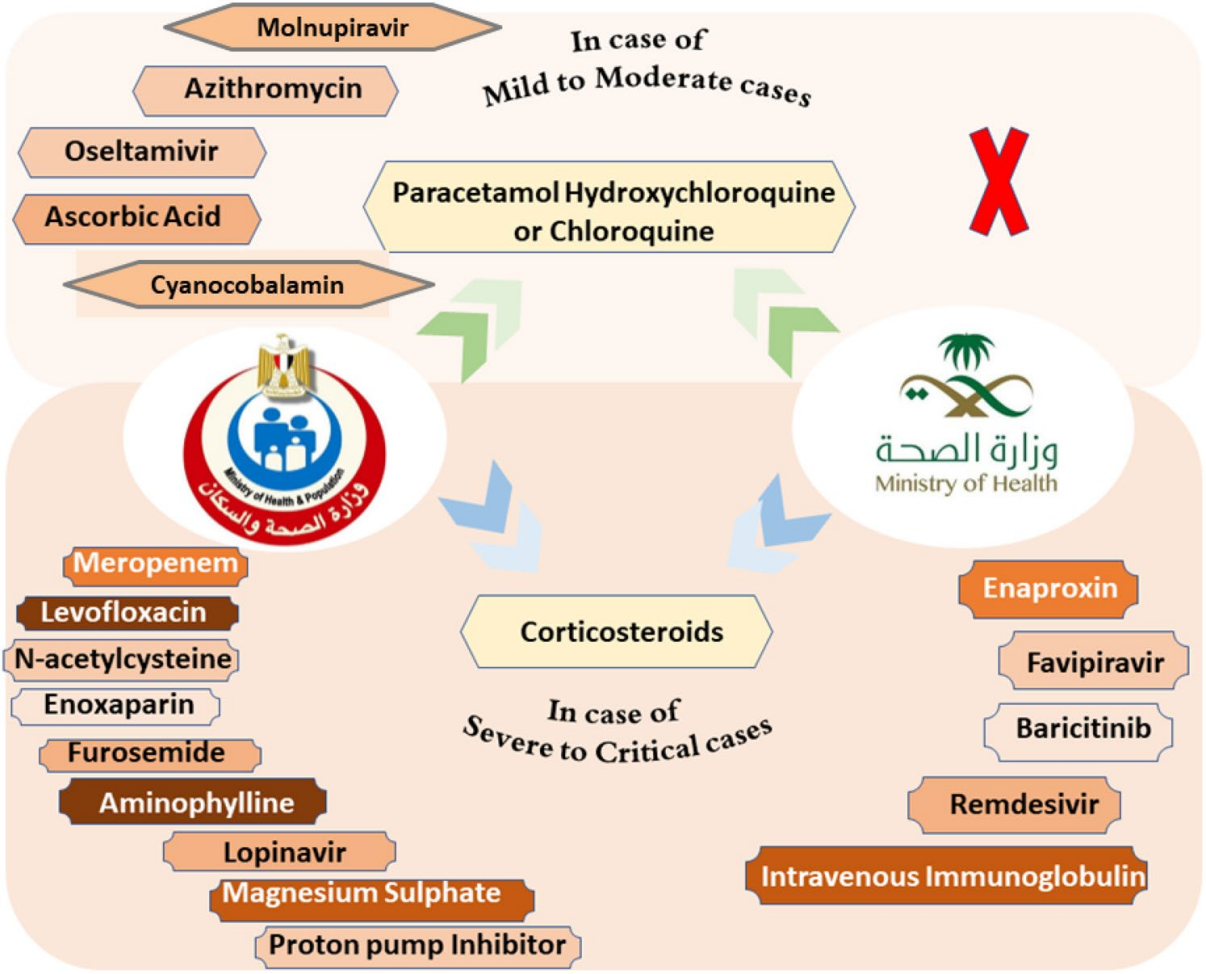
Summary of authorized drugs by the ministry of health in Egypt and the Kingdom of Saudi Arabia managing the various symptoms of COVID-19

In addition to the supportive treatment, including bed rest, preserving hydration and electrolyte balance, maintaining a stable environment, closely monitoring vital signs and oxygen saturation, providing drugs with potential antiviral activity (Shang et al. 2020). These drugs differ from the approved protocols by the ministry of health in each country as summarized in Figure 2 and Table 3.

**Table 3:** List of authorized drugs utilized in COVID-19 treatment protocols in Egypt and the Kingdom of Saudi Arabia based on the degree of disease severity

Degree of severity	Protocol of Egypt*	Protocol of Kingdom of Saudi Arabia*
Mild to moderate	-**Paracetamol,** -**Hydroxychloroquine or Chloroquine**, -Azithromycin, -Oseltamivir, -**Molnupiravir** -Ascorbic Acid, and -Cyanocobalamin	-**Paracetamol**, and -**Hydroxychloroquine or Chloroquine** -**Molnupiravir** -ritonavir-boosted nirmatrelvir (Paxlovid) -Budesonide -Anti-SARS-CoV-2 monoclonal antibodies
Severe and critical	Drugs used in mild to moderate plus the followings	
	-*N*-Acetylcysteine, -Meropenem, -Levofloxacin, -Magnesium Sulphate, -Aminophylline, -Enoxaparin, -**Corticosteroids**, -Proton Pump Inhibitor, -Furosemide, and -Lopinavir -Monoclonal antibodies	-Remdesivir, -Favipiravir, -Baricitinib, -**Corticosteroids**, -Enaproxin, -Intravenous Immunoglobulin (IVIG) -Tocilizumb (Anti-SARS-CoV-2 Monoclonal Antibody)

*Bolded words represent the drugs that are used in both Egyptian and the Kingdom of Saudi Arabian protocols

It is believed that two main processes drive COVID-19 pathogenesis. In the early stage of the clinical course, the disease is mainly driven by the replication of SARS-CoV-2. Later, the disease seems to be driven by a dysregulated immune and/or inflammatory response resulting in tissue damage. Thus, antiviral therapies would have the highest positive outcome when the course of the disease is in its early stage, while the immunosuppressive and/or anti-inflammatory treatments are more likely to be useful in the later stages of the clinical course of COVID-19 (Trougakos et al. 2021).

In the following subsections, each authorized drugs would be covered following its pharmacological class, in addition to the importance and mechanism of action, into antiviral, antimicrobial, anti-inflammatory, antioxidant, and miscellaneous.

### Antiviral drugs

**Fig. 3:**
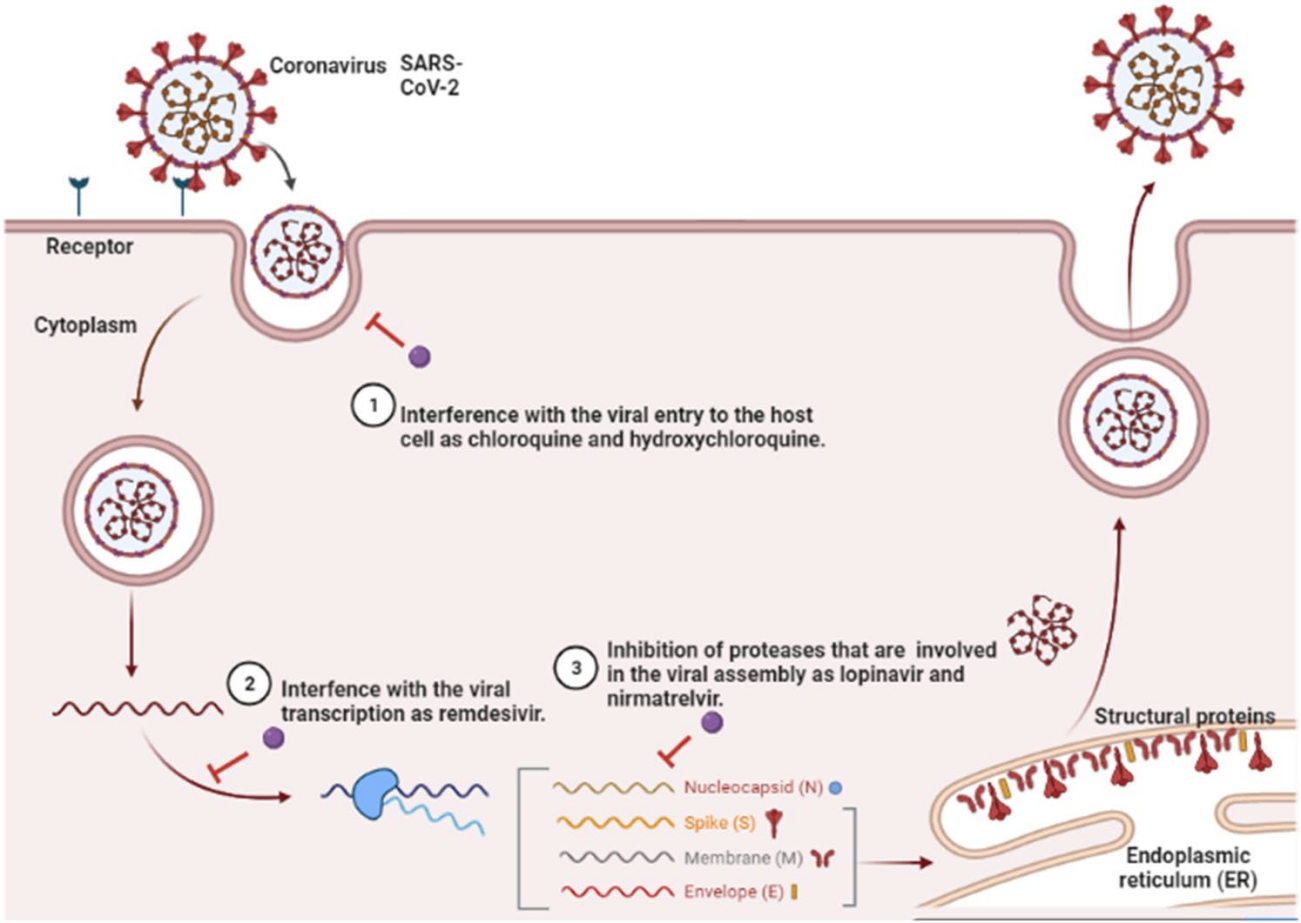
Potential mechanisms of the antiviral drugs against the SARS-CoV-2 targeting viral entry, transcription, and assembly. Antivirals as chloroquine and hydroxychloroquine can act on the viral entry, while remdesivir on the transcription step and lopinavir as well as nirmatrelvir on inhibition of viral proteases

The antiviral drugs act *via* different modes interfering with either viral entry, viral transcription, or inhibition of proteases that are involved in viral assembly, Figure 3.

#### Hydroxychloroquine and chloroquine

Chloroquine and hydroxychloroquine (HCQ) were suggested to be beneficial against SARS-COV-2. This is based on the *in vitro* finding showing that both drugs could cause glycosylation of ACE2 receptors making cells refractory to infection by SARS-COV-2 (Pahan and Pahan 2020) Although, the use of hydroxychloroquine did not result in a substantially reduced or increased risk of intubation or death in COVID-19 patients (Geleris et al. 2020). Moreover, both drugs were revealed to have immunomodulatory effects and thus potentially helpful in the reduction of COVID-19 severity (Ghazy et al. 2020; Schrezenmeier and Dörner 2020). Unfortunately, there are many controlled trials that revealed that both hydroxychloroquine and chloroquine have little or no value for people infected with COVID‐19 especially in decreasing the risk of death and might be with no effect on the progression to mechanical ventilation. These results could mean that these drugs are less likely to be effective in protecting people from COVID-19 infection. Although most of the management protocols have not recommended the use of chloroquine and hydroxychloroquine, it is believed to be effective in patients with resistant fever and to have a synergistic effect with azithromycin. One of the side effects of chloroquine is gastric upsets, so proton pump inhibitors (PPI) are to be described. Chloroquine is contraindicated in pregnant, hepatic, and cardiac patients (Hoffmann et al. 2020; Han et al. 2021).

#### Oseltamivir

Oseltamivir is a neuraminidase inhibitor that have FDA approval in 1999 (Hayden et al. 1999). Since that time, it has played a vital role in the treatment of both influenza A and influenza B infections (Zhang and Yap 2004) by preventing the release of virions from the infected cells and significantly hindering their spread in the body (Ward et al. 2005). Even though SARS-CoV-2 does not express neuraminidase, Zhang et al. discovered through homology modelling that the Spike 1 (S1) protein of SARS-CoV has an active core like that of neuraminidase. In other words, by focusing on S1 protein activity, neuraminidase inhibitors could prevent SARS-CoV from spreading. (Zhang and Yap 2004). A research group has explored the effect of oseltamivir in silico, suggesting that it is highly effective against SARS-CoV-2 especially if combined with Lopinavir/Ritonavir (Muralidharan et al. 2021). However, other studies have debated these results disapproving the use of oseltamivir in COVID-19 treatment (Rosa and Santos 2020; Tan et al. 2020).

#### Remdesivir

Remdesivir is an inhibitor of the viral RNA-dependent RNA polymerase leading to pre-mature termination of the viral RNA transcription and subsequently results in RNA synthesis inhibition (Khanal 2020; Saha et al. 2020). It was found to have an *in vitro* inhibitory activity against SARS and MERS-CoV (Brown et al. 2019; Sheahan et al. 2020). It was early identified as an intriguing candidate for COVID-19 treatment owing to its *in vitro* capability to inhibit SARS-CoV-2 and this was proved by several researchers who conducted clinical trials and they found that remdesivir resulted in a shorter time to recovery of COVID-19 patients. Monitoring of liver and kidney functions is recommended as when they increase, remdesivir should be stopped (Spinner et al. 2020; Wang et al. 2020a, b, c).

#### Lopinavir

Lopinavir is a protease inhibitor that is approved to be used for human immunodeficiency virus 1 treatment as it can resemble the peptide linkage and bind to the substrate-binding pockets of the virus enzymes. Thus, inhibiting the enzyme activity and resulting in the formation of immature and non-infectious viral particles (Uzunova et al. 2020). Previous trials and *in vitro* studies on coronaviruses including SARS and MERS have reported that lopinavir, especially when administered with ritonavir to enhance its plasma half-life, conferred clinical benefits (Li et al. 2020; Yao et al. 2020). This is attributed to its ability to inhibit the main protease of coronaviruses, which is critical for virus replication (Liu and Wang 2020). Thus, many studies have been conducted to evaluate the usefulness of lopinavir in the treatment of COVID-19 patients. One of these studies has found that lopinavir reduced the clinical symptoms in the treated animals with no effect on the virus titers (Park et al. 2020a, b). Other observational studies have reported that lopinavir decreased the duration of virus shedding (Yan et al. 2020) and fever (Ye et al. 2020).

#### Favipiravir

Favipiravir, an anti-RNA virus synthetic prodrug, has been approved in Japan for the treatment of the emerging pandemic influenza infections in 2014 (Furuta et al. 2013). Within the tissue, it does phosphoribosylation to the active drug that acts as a substrate for the RNA-dependent RNA-polymerase enzyme of the virus, thus decreasing its activity and resulting in the termination of the viral protein synthesis (Madelain et al. 2016). Some clinical studies have been conducted worldwide to evaluate the effectiveness of favipiravir in COVID-19 management as it is considered a promising candidate for the treatment of such pandemic infection due to its ability to inhibit RNA-dependent RNA-polymerase of SARS-CoV-2 (Shannon et al. 2020). These trials revealed that favipiravir has resulted in a shorter length of time of viral clearance (Cai et al. 2020) and enhanced clinical improvement (Agrawal et al. 2020).

#### Molnupiravir

Molnupiravir is an oral prodrug of *β*-_D_-N4-hydroxycytidine (NHC), a ribonucleoside analog that has broad antiviral activity against RNA viruses. NHC uptake by viral RNA-dependent RNA-polymerases results in viral mutations and lethal mutagenesis. It has strong antiviral properties against SARS-CoV-2 (Fischer et al. 2021a; Kabinger et al. 2021; Zhou et al. 2021) with good safety and tolerability profile (Fischer et al. 2021b). It was issued on 23/12/2021 by FDA for treatment of mild to moderate COVID-19. It was approved to be used within 5 days of symptom onset, for patients who lack an alternative to alternate antiviral medications and are at high risk of developing a severe illness. In the MOVe-OUT trial, molnupiravir decreased the rate of hospitalization or death by 30% relative to placebo (Singh et al. 2022). There is a certain concern, theoretically, regarding that molnupiravir might be incorporated into the host DNA cells, resulting in different mutations. Till now, there is no detected risk for genotoxicity depending on the available information introduced to FDA (Singh et al. 2022).

#### Ritonavir-boosted nirmatrelvir (Paxlovid.®)

Nirmatrelvir is an antiviral drug against all coronaviruses including COVID-19. It inhibits viral protease enzyme (MPRO), It is co-administered with ritonavir as Paxlovid. Ritonavir is a potent CYP 3A4 (cytochrome P450) inhibitor and could boost HIV protease inhibitors. This combination was authorized by FDA on 22/12/2021 for COVID-19 treatment (Pillaiyar et al. 2016; Owen et al. 2021; Saravolatz et al. 2022). It is prescribed for mild to moderate cases of COVID-19 who are at high risk to turn into severe cases. Paxlovid was expected to treat Omicron (B.1.1.529) variant and its BA.2 subvariant (Takashita et al. 2022). 89% reduction of risk of hospitalization or death according to placebo was reported in patients of SARS-CoV-2 (Hammond et al. 2022; Saravolatz et al. 2022)It showed a greater effect than remdesivir (87% relative reduction) (Gottlieb et al. 2022) and molnupiravir (30% relative reduction) (Jayk Bernal et al. 2022).

### Antimicrobial drugs

#### Azithromycin

It has been shown that azithromycin (an antibacterial macrolide compound) has an *in vitro* and *in vivo* antiviral activity against a large panel of viruses including Ebola, Zika, influenza H1N1 virus, respiratory syncytial virus, enterovirus, and rhinovirus (repurposing of azithromycin to be used as antiviral agent) (Beigelman et al. 2015; Retallack et al. 2016; Mosquera et al. 2018; Wu et al. 2018; Li et al. 2019; Tran et al. 2019; Du et al. 2020). Also, it has been found that it has a significant antiviral effect against SARS-CoV-2 (Zeng et al. 2019; Andreani et al. 2020; Bleyzac et al. 2020; Gautret et al. 2020). Azithromycin is supposed to decrease the SARS-CoV-2 virus's entrance into human cells (Lythgoe and Middleton 2020; Yuki et al. 2020). Moreover, it can modulate the immune response of the body via suppressing several cytokines that are involved in the respiratory syndrome of COVID-19 patients. It decreases the production of several pro-inflammatory interleukins (IL) including IL-1β, IL-6, IL-8, IL-10, IL-12, and IFN-α (Chen et al. 2020a, b; Huang et al. 2020; McGonagle et al. 2020). Another important property of azithromycin is its antibacterial properties, which would prevent or treat secondary bacterial infections in COVID-19 cases (Bleyzac et al. 2020).

#### Meropenem

Meropenem is a carbapenem antibacterial compound with a broad spectrum of activity including Gram-positive, Gram-negative, and anaerobic bacteria (Morris and Cerceo 2020). Like all other β-lactams, meropenem inhibits the synthesis of bacterial cell walls via binding to transpeptidases or penicillin-binding proteins (PBPs) leading to their inactivation (Townsend et al. 2020). It is prescribed in COVID-19 to treat both communities acquired, and hospital-acquired pneumonia (Rawson et al. 2020). Further, it is crucial in the treatment of patients suffering from secondary bacterial co-infections that are not linked to their respiratory presentation such as bloodstream or urinary tract infections. Meropenem is recommended in patients with secondary bacterial infections or immunocompromised patients (Hughes et al. 2020; Rawson et al. 2020; Seaton et al. 2020).

#### Levofloxacin

Levofloxacin belongs to fluoroquinolones which are synthetic broad-spectrum antimicrobial agents (Pham et al. 2019). Remarkably, levofloxacin could exert an antiviral action against many viruses such as vaccinia, cytomegalovirus, herpes simplex, varicella-zoster, hepatitis C, and human immune deficiency viruses (Khan et al. 2012; Karampela and Dalamaga 2020; Scroggs et al. 2021). It could also suppress the replication of SARS-CoV-2 via binding to its main protease. Interestingly, it exhibits an immunomodulatory activity via inhibition of the pro-inflammatory cytokines leading to attenuation of the inflammation response (Karampela and Dalamaga 2020). Notably, levofloxacin is significant in the management of severe community-acquired pneumonia due to its pharmacokinetic properties (Noreddin and Elkhatib 2010), thus it is important in the treatment of pneumonia in COVID-19. Levofloxacin is contraindicated with chloroquine in patients with heart diseases, and its dose must be adjusted in patients with renal diseases (Noreddin and Elkhatib 2010; Khan et al. 2012; Karampela and Dalamaga 2020; Scroggs et al. 2021).

### Anti-pyretic and anti-inflammatory drugs

#### Paracetamol

In March 2020, non-steroidal anti-inflammatory drugs (NSAIDs) were strongly discouraged to be prescribed to COVID-19 cases owned to the alert by several studies (Micallef et al. 2020a, b) on the possible aggravation of the disease and the risk of secondary infections and complications, mainly of the lungs. In addition, ibuprofen especially induces overexpression of angiotensin-converting enzyme 2 (ACE2) which helps the primary entry of SARS-CoV-2. However, paracetamol is considered a safer antipyretic alternative for the early management of both pain and fever in COVID-19 patients having neither anti-inflammatory nor antiplatelet activity (Driver et al. 2020).

#### Baricitinib

It has been speculated that mitigation of the immune response and prevention of the hyperinflammatory state in COVID-19 patients could improve clinical outcomes. Baricitinib is a selective inhibitor of Janus kinase (JAK) 1 and 2 and it has a therapeutic application in the treatment of inflammatory diseases as rheumatoid arthritis (Schwartz et al. 2017). It was predicted to have potential therapeutic activity against SARS-CoV-2 (Richardson et al. 2020; Stebbing et al. 2020). Baricitinib blocks the cytokine signalling pathway that is activated in COVID-19 severe instance, including IL-2, Il-6, IL10, and interferon-γ, therefore, it effectively suppresses the cytokine storm (Kalil et al. 2021; Sims et al. 2021).

### Antioxidant drugs

#### Ascorbic acid (vitamin C)

Vitamin C has various potentials which make it a beneficial medicinal agent for different respiratory infections such as COVID-19 and influenza. It has a strong antioxidant, anti-inflammatory, and immune-supportive compound (Hoang et al. 2020). Vitamin C has an important role in virus infection via dissipation of the pro-inflammatory response, improvement of the function of the epithelial barrier, enhancement of the alveolar fluid clearance, and inhibition of the sepsis-associated coagulation abnormalities (Bharara et al. 2016). Moreover, vitamin C has a significant impact on the production of type I interferons attenuating the immune response against viruses (Kim et al. 2013) and it can upregulate the activity of natural killer cells and cytotoxic T-lymphocyte both *in vitro* and *in vivo*. A common side effect of vitamin C is gastric upset, so PPIs are recommended (Carr and Maggini 2017).

#### Cyanocobalamin

Cyanocobalamin is a synthetic product of vitamin B12. Vit. B12 has an effective role in the red blood cells and myelin synthesis in addition to its important function in cellular growth. Also, it is a modulator for gut microbiota and its low levels could result in elevation of methylmalonic acid and homocysteine, leading to an increase in inflammation and oxidative stress (Mikkelsen et al. 2017). Nevertheless, SARS-CoV-2 could interfere with the metabolism of vitamin B12 causing symptoms of vitamin B12 deficiency which might lead to diseases of the gastrointestinal, respiratory, and central nervous systems. Besides, vitamin B12 deficiency results in elevated oxidative stress, activation of the coagulation cascade, vasoconstriction, pulmonary and renal vasculopathy (Sabry et al. 2020).

#### *N*-acetyl cysteine

*N*-acetyl cysteine is a precursor of the antioxidant glutathione with a direct effect on many oxidant species. It also exhibits mucolytic activity as it can loosen the thick mucus in the lungs. Moreover, it has a role in boosting the immune system and reduction of inflammation (McCarty and DiNicolantonio 2020). The E protein of SARS-CoV-2 has a triple cysteine motif that interacts with a similar motif in the S protein through disulfide bonds, and N-acetyl cysteine can cleave these bonds thus, it would decrease SARS-CoV-2 infectivity (Jorge-AarÃ3n and Rosa-Ester 2020).

### Miscellaneous drugs

#### Magnesium sulphate

Electrolytes such as sodium, potassium, and magnesium are basic elements for the maintenance of normal cell physiological function (Palmer and Clegg 2016, 2019). Magnesium is mainly concentrated in the mitochondria, and it is an essential element for various biochemical reactions in the body in addition to its participation in certain physiological functions and metabolism (Komiya and Runnels 2015), such as energy metabolism and synthesis of both proteins and nucleic acids (Abiri and Vafa 2020). Magnesium also has anti-inflammatory and antioxidant activity (Han et al. 2018; Abiri and Vafa 2020; Ozen et al. 2020) and it is vital in the regulation of homeostasis of different systems such as the digestive, neurological, and respiratory systems (Tang et al. 2020; Bachnas et al. 2021). Using the aforementioned data, it is proved that magnesium sulfate can be beneficial in the supportive cure of COVID-19 especially in critically ill patients (Tang et al. 2020).

#### Aminophylline

Aminophylline is a drug made of theophylline and ethylenediamine with a ratio of 2 to 1. It is approved by FDA to be used in relieving symptoms of reversible airway blockage as in asthma or other chronic lung diseases like chronic bronchitis (Armanian et al. 2014). Its mechanism of action is not completely understood but it seems like the smooth muscle relaxation of the lungs and pulmonary way via phosphodiesterase inhibition (Rabe et al. 1995). It is also an adenosine receptor antagonist; thus, it has an indirect role in bronchodilation (Polosa and Blackburn 2009), and theophylline increases the activity of histone deacetylases to the site of active inflammation leading to the anti-inflammatory effect (To et al. 2010; Wei et al. 2019). Aminophylline releases theophylline, after administration, which is responsible for bronchodilation.

#### Enoxaparin

Enoxaparin is low molecular weight heparin and was first approved for medical use in the treatment of venous thromboembolism in 1993 (Miranda et al. 2017; van Gameren et al. 2018; Xing et al. 2018). It potentiates antithrombin III to form a complex that leads to irreversible inactivation of factor Xa (Nutescu et al. 2016). It is thought that enoxaparin has a potential therapeutic impact in COVID-19 patients. This is because enoxaparin plays a part in preventing COVID-19 infection via decreasing the viral entry to human cells. It can also reduce the release of IL-6 that is associated with cytokine storms. In addition to its ability to prevent the activation of the coagulation cascade, venous thromboembolism, and thrombosis in the small and middle-size vessels of the lung (Drago et al. 2020; Hasan et al. 2020). The use and dose of enoxaparin are decided following the D-dimer test. Anticoagulants in COVID-19 Patients with an elevated D-dimer are recommended. Daily monitoring of PT/INR and PTT is also advised for patients concurrently on enoxaparin and used with precautions in hypertensive patients (Organization 2020a, b; Han et al. 2021).

#### Corticosteroids

Corticosteroids include glucocorticoids and mineralocorticoids, and they are produced by the adrenal cortex. They do not attack the viruses directly, rather they act through their immunosuppressive and anti-inflammatory activities (Ramamoorthy and Cidlowski 2016). Their anti-inflammatory activity is attributed to the suppression of the pro-inflammatory genes via signal transduction by binding to their steroid receptors (Cruz-Topete and Cidlowski 2015; Budhathoki et al. 2020). Due to their effects, corticosteroids have been used in the past during the outbreaks of SARS-CoV and MERS-CoV (Russell et al. 2020), and their uses in the current days in the pandemic of COVID-19 is based on the genetic homology with both SARS and MERS coronaviruses (Budhathoki et al. 2020). There is a controversy regarding the use of corticosteroids in COVID-19 patients. Wu and his colleagues noticed a reduction in mortality when used in COVID-19 patients with acute respiratory distress (Wu et al. 2020), whereas many other retrospective studies revealed an increase in mortality among the corticosteroid receiving groups. Corticosteroids are not used in the first days of infection unless with hypoxic patients or patients with severe pulmonary abnormalities on chest X-ray. Diabetic patients on corticosteroids are closely monitored, and gradual steroid withdrawal is needed to avoid sudden termination and unwanted consequences (Huang et al. 2020; Wang et al. 2020a, b, c; Zhou et al. 2020).

#### Proton pump inhibitors

Proton pump inhibitors are drugs for treating gastric acid-related diseases by suppressing the acid secretion in the gastric lumen via inhibiting H^+^/K^+^ ATPase. Additionally, it was revealed that the proton pump inhibitors are effective on the elements of the immune system including neutrophils, monocytes, and endothelial cells (Wandall 1992). They can suppress the functions of neutrophils such as chemotaxis and superoxide production (Ubagai et al. 2009). Moreover, they have an anti-inflammatory effect through the reduction of the neutrophil adhesion molecules and free oxygen radicals (Biçakçi et al. 2005). They can also activate heme oxygenase-1 (HO-1) which is an endogenous antioxidant (Taştemur and Ataseven 2020). Based on all these data plus the fact that these drugs are inexpensive, widespread, and immediately available, these drugs are used in the treatment of COVID-19 pandemic infection. In addition, proton pump inhibitors are used to relieve the side effects of other drugs involved in the management protocols.

#### Furosemide

Furosemide was approved by FDA to be used to treat diseases connected to edoema and volume overload brought on by congestive heart failure, renal failure, or liver failure (Felker et al. 2011). Its mechanism of action depends mainly on inhibition of reabsorption of sodium and chloride ions in the proximal tubules, distal tubules, and thick ascending loop of Henle via inhibition of the sodium-chloride cotransport system leading to excessive excretion of water containing sodium, chloride, calcium, and magnesium (Shankar and Brater 2003). In aged COVID-19 patients having a high proportion of cardiac comorbidities, mild and moderate pneumonia could be accompanied by a certain degree of acute heart failure and ischemia (Sisti et al. 2021). Thus, furosemide is thought to be essential for COVID-19 patients especially, when corticosteroids are administered, to limit corticosteroid-induced retention (Kevorkian et al. 2021).

#### Intravenous immunoglobulin

Polyclonal immunoglobulin gamma is the main component of intravenous immunoglobulin (IVIG), a blood product that is derived from healthy donors. In several autoimmune and inflammatory illnesses, it has been used as an immunomodulatory treatment. (Galeotti et al. 2017). Remarkable positive results have been perceived by the administration of IVIG to patients with SARS and MERS (Arabi et al. 2014). Bearing in mind the devastating immune response that occurs in many COVID-19 patients (Zhu et al. 2020), in addition to the similarities in pathogenesis between SARS and COVID-19, it seems that IVIG could produce good clinical outcomes in COVID-19 patients (Cao et al. 2020a, b). Several investigations have been carried out to assess its effectiveness in COVID-19 cases and they provided evidence that supported the IVIG injection enhances clinical results in severe COVID-19 cases (Cao et al. 2020a, b; Gharebaghi et al. 2020; Xie et al. 2020).

#### Tocilizumab (Anti-SARS-CoV-2 monoclonal antibody)

Tocilizumab is an immunosuppressive drug which is indicated in treating rheumatoid arthritis and giant cell arteritis. It is a humanized monoclonal antibody which suppresses the signalling of IL-6. IL-6 is a cytokine involved in the pathogenesis of several diseases such as lymphoproliferative and autoimmune diseases. In June 2021, it was granted for the treatment of COVID-19 as an emergency use authorization by the FDA in hospitalized patients, who are receiving systemic corticosteroids and require supplemental oxygen and non-invasive or invasive mechanical ventilation (Kenny and Mallon 2021). Tocilizumab prevented mortality in patients hospitalized for COVID-19. This effect was observed to a greater extent in patients getting concomitant corticosteroids and administered tocilizumab in the first 10 days from the begining of symptoms onset (Rubio‐Rivas et al. 2021)**.**

#### Oxygen therapy

ARDS was diagnosed in about 67 percent of patients, with 71 percent requiring artificial ventilation. For patients with SARS-CoV-2 at various stages of the disease, oxygen therapy is recommended. Oxygen saturation must be 92% or more so the need for various oxygen delivery devices is determined by the patient's condition. In mild to moderately symptomatic patients, a nasal cannula and blow-over oxygen at 4–6 L/min can be applied, with the patient's face covered with an N95 or comparable face mask. A high-flow nasal cannula delivers heated humidified oxygen at rates ranging from 10 to 50 L/min where it creates positive pressure at high flows, used especially in conditions such as respiratory distress, preoxygenation, and apneic diffusion of oxygen in airway treatments. Supraglottic devices are still recommended in severe airway situations, especially in apneic patients (https://www.covid19treatmentguidelines.nih.gov/; Lyons and Callaghan 2020; Smit et al. 2020). Common side effects of oxygen therapy are a decrease in lung secretions, dryness, middle ear barotrauma, and retinal detachment especially in pediatric patients (Heyboer et al. 2017; Barrett et al. 2020).

Drugs used in the management protocol, their doses, side effects, and contraindications are summarized in Table 4.

**Table 4:** Doses, side effects, and contraindications of drugs used in COVID-19 management protocols both in Egypt and the Kingdom of Saudi Arabia

Drug	Doses	Side effects	Contraindication	Ref
Paracetamol	500 mg/4–6 h	Gastrointestinal, hepatic, bleeding disorders	Hypersensitivity, cautions in hepatic patients	(Noronha et al. 2021)
Chloroquine (HCQ)	600 mg OD/day	Gastric upsets as vomiting and diarrhea were the common side effects. Others include dizziness, headache, dazzling, tinnitus, disturbances of taste and smell, seizures, psychosis, and irritability	Pregnant, hepatic, and cardiac patients	(Srinivasa et al. 2017)
Azithromycin	500 mg OD for 3–5 days	Gastrointestinal (nausea and abdominal pain), headache or dizziness, hepatotoxicity, and antibacterial resistance development	Hypersensitivity to any macrolide, a history of cholestatic jaundice, or hepatic dysfunction	(Echeverría-Esnal et al. 2021)
Oseltamivir	75 mg twice a day for 5 days	Gastrointestinal (nauseous, vomiting, diarrhea, stomach pain/cramps)	Hypersensitivity, children younger than one year	(Tullu 2009)
Molnupiravir	800 mg PO every 12 h for 5 days	Diarrhea, nausea, and dizziness	Pregnant, lactating persons, children	(Jayk Bernal et al. 2022)
Ascorbic acid,	500 mg to 1 g OD/day	Headaches, flushing, nausea or vomiting, and dizziness	Blood disorders	(Abdullah et al. 2020)
Cyanocobalamin	1-2 mg OD/day	Allergic reactions and other rare effects as ever, itching or rash, tingling or numbness of joint, shortness of breath	Hypersensitivity cautions in nerve atrophy, renal patients	(Al Amin and Gupta 2020)
N-acetylcysteine	200-600 mg/day	Gastrointestinal (nausea, vomiting, diarrhea, flatus)	Congestive heart failure or patients with a tendency to develop edema	(Ershad and Vearrier 2019)
Meropenem	3 to 6 g IV/day	Increase the risk of developing neurotoxicity and nephrotoxicity	Hepatic, renal dysfunction, and pregnant	(Steffens et al. 2021)
Levofloxacin	750 mg for 5 days	Photosensitivity, nausea, diarrhea, headache, tendinitis, tendon rupture, hyper-hypoglycemia, seizures, and peripheral neuropathy	Hypersensitivity, pregnancy, nursing mothers, and in children younger than 18 years due to possible risk of cartilage damage	(Podder and Sadiq 2019)
Magnesium sulphate	4 g IV/day	Nausea, vomiting, headache, and palpitations. hypotension and respiratory depression	Respiratory arrest and cardiac arrest	(Tang et al. 2020; Bachnas et al. 2021)
Aminophylline	400 to 600 mg/day	nausea, vomiting, headache, increase in urine volume, insomnia, irritability, restlessness	Cardiac, Renal, hepatic dysfunction, hypo /hyperthyroidism, epilepsy	(Zafar Gondal and Zulfiqar 2019)
Enoxaparin	1 mg/km every 12 h	Nausea, headache, bleeding, rectal sheath hematoma, liver injury	Hepatic diseases, ulcers, bleeding, uncontrolled hypertension, hemophilia, thrombocytopenia	(Jupalli and Iqbal 2020)
Corticosteroids	5 to 60 mg OD/day	Shock, anorexia, headache, fever, joint pain, hypotension, nausea, and vomiting, hyperglycemia, Myopathy, Glaucoma	Hypersensitivity, uncontrolled hyperglycemia, diabetes mellitus, glaucoma, joint infection, uncontrolled hypertension	(Hodgens and Sharman 2020; Langarizadeh et al. 2021)
Proton pump inhibitor	20 to 40 mg OD/once or twice a day	Iron deficiency, hypomagnesemia, increased risk of fractures, pneumonia, enteric infections, hypergastrinemia, and cancer	Hypersensitivity, blood disorders, hepatic dysfunction	(Biçakçi et al. 2005; Ubagai et al. 2009; Taştemur and Ataseven 2020)
Furosemide	40 mg to 80 mg OD/day	Hypokalemia, hypomagnesemia, hypocalcemia, hyperglycemia, glycosuria, hyperuricemia, hypertriglyceridemia, increased cholesterol levels, orthostatic hypotension	Hypersensitivity, anuria	(Khan et al. 2021)
Lopinavir	400 mg twice a day	Diarrhea, nausea, asthenia; elevations in total cholesterol, triglyceride, and AST/ALT level	Hypersensitivity, pregnancy (Category C)	(Cao et al. 2020)
Paxlovid	300 mg nirmatrelvir with 100 mg of ritonavir twice daily for 5 days	impaired sense of taste, diarrhoea, vomiting and headache	liver disease, liver enzyme abnormalities	(Duffy 2022)

## In comparison to the management protocol of the United States of America and the rest of the world

The line of treatment and drugs administered in COVID-19 management protocols are quietly similar worldwide. In the USA, however, mild and moderate cases of COVID-19 should be administered with anti-SARS-CoV-2 antibody-based therapies including bamlanivimab plus etesevimab or casirivimab plus imdevimab. Bamlanivimab is a neutralizing monoclonal antibody that can target the receptor-binding domain (RBD) of the spike (S) protein of SARS-CoV-2. Etesevimab is another neutralizing monoclonal antibody that can bind to a different and overlapping epitope in the S protein RBD of SARS-CoV-2. Both casirivimab and imdevimab are recombinant human monoclonal antibodies that can bind to different and non-overlapping epitopes of the RBD of the S protein of SARS-CoV-2. These combination therapies are used in non-hospitalized mild to moderate COVID-19 cases with laboratory-confirmed SARS-CoV-2 infection to decrease the risk for progression to severe disease and hospitalization (https://www.covid19treatmentguidelines.nih.gov/).

Remdesivir and corticosteroids are recommended to be used in the severe cases of COVID-19 in the USA and this is like the Middle East. In the USA, an additional drug is added to the management protocol used in severe cases to improve the survival among COVID-19 patients exhibiting rapid respiratory decompensation. This drug is tocilizumab which is a recombinant humanized monoclonal antibody against IL-6 receptor (https://www.covid19treatmentguidelines.nih.gov/). IL-6 levels are highly correlated to the severity of COVID-19 suggesting that the immune dysregulation and acute respiratory distress syndrome that occur in severe cases of COVID-19 could be influenced by IL-6(https://www.covid19treatmentguidelines.nih.gov/).

The treatment guidelines for COVID-19 have many similarities among different countries worldwide. Treatment protocols usually include main four medication groups: antiviral, systematic corticosteroids, antimalarial, and antibiotics, which can vary in dosages, and durations. Notably, the cost of the utilized treatment protocols is an important factor as the prices of antimalarial drugs and corticosteroid therapy could be affordable for most of the countries. Nevertheless, the cost of the antiviral drugs and immunomodulators is very high which could hinder their availability to COVID-19 patients especially in low-income countries (https://www.covid19treatmentguidelines.nih.gov/.). The antiviral drugs used worldwide include remdesivir, lopinavir/ritonavir, darunavir/cobicistat, favipiravir, umifenovir, ribavirin, or oseltamivir. Systematic corticosteroids include dexamethasone, methylprednisolone, prednisone, prednisolone, or hydrocortisone. The conventional antimalarial drugs include hydroxychloroquine and chloroquine. Immunomodulators comprise tocilizumab, siltuximab, sarilumab, canakinumab, anakinra, ruxolitinib, or baricitinib. Aside, other medications, such as antibiotics and anticoagulants, are used in the management of COVID-19 to eradicate concurrent bacterial infections or to prevent complications (Jirjees et al. 2021). Table 5 shows a summary of different medications used for the treatment of COVID-19 patients in different countries worldwide.

**Table 5:** Summary of the medicaments used for the treatment of patients with COVID-19 in different countries

Country	Used medication
Egypt	Lopinavir /ritonavir, oseltamivir, molnupiravir, hydrocortisone, hydroxychloroquine, azithromycin, and enoxaparin
Kingdom of Saudi Arabia	Remdesivir, lopinavir /ritonavir, favipiravir, ribavirin, molnupiravir, nirmatrelvir / ritonavir, dexamethasone, methylprednisolone, prednisolone, tocilizumab, and enoxaparin
United States of America	Ritonavir-boosted nirmatrelvir, remdesivir, molnupiravir, dexamethasone, methylprednisolone, prednisone, hydrocortisone, bebtelovimab, tocilizumab, sarilumab, tofacitinib, baricitinib, and heparin
Canada	Remdesivir, Bamlanivimab, Casirivimab, imdevimab, Sotrovimab, Nirmatrelvir and ritonavir (Paxlovid™), Tixagevimab and cilgavimab (Evusheld™)
Pakistan	Remdesivir, dexamethasone, methylprednisolone, tocilizumab, azithromycin, and enoxaparin
United Arab Emirates	Remdesivir, favipiravir, dexamethasone, methylprednisolone tocilizumab, sarilumab, bamlanivimab, and enoxaparin
Belgium	Nirmatrelvir/ritonavir, remdesivir, molnupiravir, dexamethasone, methylprednisolone, tocilizumab, casirivimab, imdevimab, sortovimab, tofacitinib, baricitinib
Spain	Remdesivir, lopinavir /ritonavir, hydroxychloroquine, chloroquine, tocilizumab, siltuximab, sarilumab, ruxolitinib, baricitinib, interferon Beta-1B (IFNb) and interferon Alfa-2B, and anakinra
United Kingdom	Nirmatrelvir/ritonavir, remdesivir, molnupiravir, and sortovimab
India	Remdesivir, hydroxychloroquine, budesonide, dexamethasone, methylprednisolone, tocilizumab, and enoxaparin
Thailand	Lopinavir /ritonavir, favipiravir, hydroxychloroquine, chloroquine, and azithromycin
China	Ritonavir-boosted nirmatrelvir, lopinavir /ritonavir, darunavir/cobicistat, umifenovir, ribavirin, chloroquine, methylprednisolone, tocilizumab, and azithromycin
Singapore	Monoclonal antibodies (e.g. sotrovimab), remdesivir, nirmatrelvir/ritonavir, molnupiravir, baricitinib lopinavir /ritonavir, baricitinib dexamethasone, tocilizumab, and enoxaparin
Australia	Casirivimab plus imdevimab, sotrovimab, molnupiravir, nirmatrelvir/ritonavir, baricitinib, Tixagevimab plus cilgavimab (Evusheld), remdesivir, tocilizumab, sarilumab, baricitinib, and dexamethasone
South Africa	Paracetamol,vitamins, zinc, aspirin, anticoagulants, ivermectin, dexamethasone, prednisone, and heparin

There are many questions concerning COVID-19 that are still without clear answers owing to the disease’s novelty and the excessive load on healthcare systems. This may explain the relatively low number of publications concerning the effectiveness and safety of the endorsed drugs in the management of COVID-19 patients.

## Conclusions

COVID-19 with its spread waves has significantly affected human life since the end of 2019 with high mortality rates. Various lines of treatment have evolved due to the wide array of COVID-19 symptoms. The review evaluated authorized drugs in official protocols that were authorized in the Middle East and administered based on the degree of the disease severity. The use of such protocols has succeeded to decrease the number of hospitalized patients in two medical centers in the Middle East, *i.e.*, Egypt and the Kingdom of Saudi Arabia, despite the absence of official statistical data demonstrating the effectiveness of the used protocols. Similarities between the two countries were observed regarding the administration of corticosteroids, paracetamol, hydroxychloroquine, molnupiravir and monoclonal antibodies. Yet, the other supplementary drugs, including vitamin C and proton pump inhibitors differed in-between.

Recognizing the clinical features of COVID-19 disease is crucial to be targeted by specific therapies. For instance, the anti-viral agents could be needed to target the viral entry and replication, while the immunomodulatory drugs are more likely to have a role in the cytokine storm in patients with a high risk of requiring intensive care to prevent uncontrolled inflammation and subsequent death. Thus, immunomodulatory drugs are recommended to be added to the management protocol used in Egypt as baricitinib, which is used in the management protocol of the Kingdom of Saudi Arabia to control the cytokine storm that occurs in severe cases of COVID-19. Immunomodulatory drugs like IL-6 inhibitors include anti-IL-6 receptor monoclonal antibodies as sarilumab and tocilizumab, and anti-IL-6 monoclonal antibodies as siltuximab (https://www.covid19treatmentguidelines.nih.gov/.). The article may provide the medical staff with the required overview and compare the various used drugs to improve currently used and develop new lines of disease management and treatment, especially that the long-term effectiveness of the developed vaccines has not been confirmed yet.

## Data Availability

Enquiries about data availability should be directed to the authors.
